# The Efficacy of Probiotics as Antiviral Agents for the Treatment of Rotavirus Gastrointestinal Infections in Children: An Updated Overview of Literature

**DOI:** 10.3390/microorganisms10122392

**Published:** 2022-12-02

**Authors:** Andrej Steyer, Dušanka Mičetić-Turk, Sabina Fijan

**Affiliations:** 1 National Laboratory of Health, Environment and Food, Division of Public Health Microbiology, Grablovičeva 44, 1000 Ljubljana, Slovenia; 2 Department of Paediatrics, Faculty of Medicine, University of Maribor, Taborska ulica 8, 2000 Maribor, Slovenia; 3 Institute for Health and Nutrition, Faculty of Health Sciences, University of Maribor, Žitna ulica 15, 2000 Maribor, Slovenia

**Keywords:** probiotics, microbiota, rotaviruses

## Abstract

Enteric viruses, including the rotavirus, norovirus, and adenoviruses, are the most common cause of acute gastroenteritis. The rotavirus disease is especially prevalent among children, and studies over the past decade have revealed complex interactions between rotaviruses and the gut microbiota. One way to treat and prevent dysbiosis is the use of probiotics as an antiviral agent. This review focuses on the latest scientific evidence on the antiviral properties of probiotics against rotavirus gastroenteric infections in children. A total of 19 studies exhibited a statistically significant antiviral effect of probiotics. The main probiotics that were effective were *Saccharomyces cerevisiae* var. *boulardii, Lacticaseibacillus rhamnosus* GG, and various multi-strain probiotics. The underlying mechanism of the probiotics against rotavirus gastroenteric infections in children included immune enhancement and modulation of intestinal microbiota leading to shortening of diarrhoea. However, several clinical studies also found no significant difference in the probiotic group compared to the placebo group even though well-known strains were used, thus showing the importance of correct dosage, duration of treatment, quality of probiotics and the possible influence of other factors, such as the production process of probiotics and the influence of immunisation on the effect of probiotics. Therefore, more robust, well-designed clinical studies addressing all factors are warranted.

## 1. Introduction

Acute gastroenteritis is one of the most frequently reported infectious diseases in the world. The most common cause of acute gastroenteritis (AGE) is various enteric viruses, including rotaviruses, noroviruses, astroviruses, adenoviruses, and other less presentable viruses [1]. Most are icosahedral nonenveloped viruses, known to present stability in the environment, resistant to many physio-chemical conditions. Their stability in the environment and on various fomites is also crucial for indirect transmission via contaminated surfaces, food, and water [2]. As the infectious dose, particularly for noroviruses, is very low [3], indirect infections are possible, and each year we can follow reports on food and/or waterborne infections, mostly with noroviruses.

Rotaviruses are members of the *Reoviridae* family and are characterized by their non-enveloped, segmented, double-stranded RNA genome (11 segments). Each of the 11 genes code for a single gene product. Six of the proteins are found in the virus particle (vp1, vp2, vp3, vp4, vp6 and vp7), whereas the remaining five proteins are non-structural (NDP1–NSP5). The *Rotavirus* is classified into serogroups A to E based on antigenic properties. Only groups A to C have been shown to infect humans, and the most human *Rotavirus* disease is caused by the group A *Rotavirus*. The group A *Rotavirus* is further classified into G (serotypes) and P types based on identification of antigens on the outer capsid proteins. Group A rotavirus genotypes are classified by a nucleotide-sequence-based, complete genome classification system [4,5].

*Rotavirus* gastroenteritis is still an important public health concern. In particular, low-income countries are fighting against the rotavirus disease, especially affecting small children [6]. Rotavirus gastroenteritis is the leading global pathogen of diarrhoea-associated mortality with the highest death rate among children under 5 years worldwide. Since 2006, efficient vaccines have been available to protect children from severe rotavirus gastroenteritis [7]. However, there are still high numbers of acute rotavirus gastroenteritis in those countries.

During the post-marketing phase of rotavirus vaccines, one of the most exceptional findings was the difference in vaccine effectiveness, being much lower in low- and middle-income countries [8,9]. One of the possible explanations was the effect of histo-blood groups, which may contribute to the virus binding on these antigens [10,11]. In parallel, a new research area of the virus–bacteria interactions opened, showing that enteric viruses may bind to bacteria surface antigens, which may influence the early phases of virus pathogenesis [12,13,14]. Consequently, it is clear now that the pathogenesis of enteric viruses is dependent not only on virus pathogenetic factors or host determinants, but also on the environment. The microbiota is therefore of high importance and can influence the effectiveness of the rotavirus or other enteric virus infections. In addition, studies on probiotics are also promising in the prevention phase, and to some extent, also in the curative phase of AGE [15,16,17].

The gastrointestinal tract is one of the most microbiologically active ecosystems with a high density of bacteria and other microbes formulating the intestinal microbiota. This microbiota has several beneficial roles for its human host, including antimicrobial activity, competitive exclusion, immunomodulation, strengthening of the epithelial barrier function, as well as influencing the immune system, central nervous system, and endocrine system [18,19,20,21]. Recent evidence-based research shows that the gut microbiota is an ally for the interaction with most human cells via the microbiota-gut-brain axis, microbiota-gut-skin axis, microbiota-gut-lung axis, microbiota-gut-liver axis, microbiota-gut-vagina axis, and many more axes. The microbiota thus aids in achieving homeostasis of skin health, respiratory health, organ health, mental health, and so forth of its host [19,20,21,22,23,24,25,26,27,28]. The intestinal microbiota coexists with microbes that reach the intestine through food intake and influences the immune cells associated with the lamina propria through the production of metabolites, crucial for the maturation of immune cells in the mucosal immune system [19,21,29]. Disruption of the homeostasis between the intestinal microbiome and the host immune system can adversely impact viral immunity [30].

Rotaviruses infect the small intestine, an important site of colonization by the microbiota, and studies over the past decade have begun to reveal a complex set of interactions between rotaviruses and the gut microbiota, as rotavirus infection can temporarily alter the composition of the gut microbiota [13]. One way to treat and prevent dysbiosis is the use of probiotics. Probiotics are, by definition, “live microorganisms that, when administered in adequate amounts, confer a health effect on the host” [31]. Scientific evidence shows enough evidence to justify the use of probiotics for the treatment of several disorders, including gastrointestinal dysbiosis, antibiotic-associated diarrhoea, irritable bowel syndrome, and inflammatory bowel disease, as well as anxiety, depression, and wound healing [19,20,32,33,34,35]. In a review on the management of acute gastroenteritis in Jordanian children [36], it was emphasised that prevention of diarrhoea diseases should focus on the improvement of nutrition, hygiene, and sanitation. In the case of rotavirus gastroenteritis, the authors proposed the introduction of routine vaccination against the rotavirus, as well as the use of adjuvant therapies. One of these possible therapies is probiotics. Other reviews addressing gastrointestinal infections also conclude that probiotics are one of the possible adjuvant strategies for diarrhoea in children by resuming a healthy microbiota status following infection [37,38,39] A recently published review even suggested the potential of a combined lactic-acid bacteria vaccine as an alternative recombinant vaccine against the rotavirus [40]. Two reviews have already addressed the efficacy of using probiotics for rotavirus infection in children, one published in 2015 [41] and another published in 2020 [42]. The review from 2015 focussed on the duration of rotavirus diarrhoea in children, whilst the 2020 review found that probiotics could reduce the occurrence of acute rotavirus diarrhoea in children. Our review investigated the underlying antiviral mechanisms of probiotics against rotavirus infections in children, includes updated information, and focused on the effective mechanisms of probiotics.

## 2. Rotavirus Infection and the Gut

The *Rotavirus* infects the mature enterocytes in the middle and upper parts of the villi and in the enteroendocrine cells in the small intestine, which ultimately leads to diarrhea [43]. Rotavirus infection can temporarily alter the composition of the gut microbiota, thus leading to dysbiosis [13]. According to one study, dysbios is caused by a decrease in the amount of bifidobacteria, normal *Escherichia coli,* and an increase in the amount of lactose-negative *Escherichia*. In cases of pronounced dysbiosis in young children, the clinical course of rotavirus infection is aggravated and the period of rotavirus excretion is prolonged [44]. Other studies found that patients with diarrheal stools with rotavirus had more bacterial communites at the genus level containing specific diarrheal causative bacteria than those of healthy subjects, suggesting that co-infection with the virus and bacteria could have occurred in some diarrhea cases [43]. Gut dysbiosis due to viral infection could be associated with a reduction in the populations of common and beneficial bacterial species and the resulting loss of diversity, as well as the gain of harmful bacteria. It may also be due to variations in crosstalk via direct interaction between rotaviruses and bacteria in the gut [43,45].

A symptomatic infection with rotaviruses stimulates a strong humoral IgG immune response which lasts for a lifetime. While the IgG responses are easily recorded, it is generally thought that protection from rotavirus disease is mediated by local IgA antibodies [4,46].

## 3. Probiotics and the Antiviral Mechanisms

Probiotic administration stimulates the immune system by inducing a network of signals mediated by various metabolites. Some probiotic strains stimulate the immune response and are therefore beneficial for patients suffering from immune deficiency, whilst other strains inhibit the immune response and are therefore beneficial for patients with conditions with immune activation. Additionally, the effects of probiotic modulation on the immune cells can be observed in lymphocytes, hematopoietic stem cells, T cells, macrophages, natural killer cells, and dendritic cells. Additionally, molecules usually associated with pathogens, such as lipopolysaccharide of gram-negative bacteria or lipoteichoic acids of gram-positive bacteria, can be produced by probiotics and interact with different toll-like receptors, and incite NF-κB-mediated antiviral gene expression [19,34,35,47,48].

It is also known that respiratory viruses can cause changes in the gut microbiome, therefore probiotics are a possible medication to treat respiratory viral infections via gut-microbiota modulation and production of immunomodulatory agents. Interactions between probiotics, macrophages, and dendritic cells are seen in the lamina propria, resulting in natural killer (NK) cell activation, which triggers interferon gamma (IFN-γ) production to defend against viruses, and efficient immune cells go to infection sites via circulatory and lymphatic systems to protect against respiratory viruses [35,47,48].

Bacteriocins produced by probiotics have also proven effective against viral infections as they exhibit antimicrobial potential against viral pathogens by prevention of viral particle aggregation and blocking the sites of host cell receptors or inhibition of viral penetration into human cells [49,50,51,52].

All above-mentioned mechanisms collectively lead to the indirect consequence of a shorter infectious period and overall reduction in the risk of viral infection [53,54,55]. 

On the other hand, previous bacterial infections in children may increase the risk of rotavirus infections by disrupting the balance of the intestinal microbiota, leading to dysbiosis and increasing the ratio of pathogenic bacteria [56,57]. Co-infection with bacterial diarrhoea-related bacterial pathogens, such as *Escherichia, Shigella, Klebsiella,* and *Campylobacter* spp., can cause a more severe course of the rotavirus disease [43]. Although it is well-established that probiotics display antibacterial activities against common pathogenic bacteria, including competitive exclusion, bacteriocin production, enhancing intestinal barrier function, and stimulation of host antimicrobial defences [46], these bacterial infections can antagonise the antivirus effects of probiotics while they are fighting off bacterial pathogens. Therefore, more research into the complex mechanisms of actions of probiotics and pathogens is warranted.

## 4. Clinical Trials with Established Antiviral Effect of Probiotics against Rotaviruses

We used the search strategy: “probiotics” AND rotavirus in various databases (PubMed, Web of Science, Scopus) and included clinical trials, which found a statistically significant antiviral effect of probiotics in the treatment of rotavirus gastroenteritis. Clinical trials without the full text available and in languages other than English were excluded. Clinical trials where rotaviruses were not determined or detected were also excluded. A total of 19 clinical studies with a statistically significant antiviral effect of probiotics against rotaviruses were found. These studies were conducted in Argentina, Bangladesh, Bolivia, Brazil, Croatia, Denmark, Egypt, Greece, India, Israel, Italy, the Republic of Korea, the Netherlands, Poland, Portugal, Slovenia, Taiwan, Turkey, and the United Kingdom. The characteristics of the clinical trials are described in Table 1.

Our review included two additional clinical trials [58,59] compared to the 2020 review [42] and several more compared to the 2015 review [41] which selected clinical trials published until the year 2013. Strain-specific antiviral activity of probiotic strains, as well as the concentration of probiotic supplements and duration of supplementation, seem to be the most important factors that influence the efficiency of probiotics on rotavirus disease in children [55].

Rotaviruses can cause significant diarrhoeal disease in infants and young ones of various mammalian and avian species [15]. According to European Society for Paediatric Gastroenterology, Hepatology and Nutrition/ESPGHAN/ESMAD, the standard recommended treatment for acute diarrhoea in children, whether due to the rotavirus, norovirus, bacterial or other infection, includes oral rehydration solutions (ORS) and continuance of feeding. Adjuvant therapy with micronutrients, probiotics, or anti-diarrhoea agents are also rendered useful. The recommended probiotics are *Lacticaseibacillus rhamnosus* GG (ATCC 53103), also known as LGG, and the yeast *Saccharomyces cerevisiae* var. *boulardii* [77,78,79,80].

The underlying mechanism against rotavirus infections is immune enhancement, as certain strains of lactobacilli promote immunological responses. This includes increasing concentrations of anti-rotavirus-specific IgA [55,81], reducing intestinal microbiota imbalance, enhancing the colonization of probiotics [82,83], and reducing the incidence of diarrhoea [84]. One important activity of probiotics is also increasing the clearance of stool rotavirus by reducing faecal rotavirus shedding, and thus aiding the epidemiological importance in the transmission of rotaviruses [85,86].

The beneficial effects of probiotics in the 19 studies noted in Table 1 have confirmed an antiviral effect of certain probiotics, leading to shortening of diarrhoea in children due to rotavirus enteritis after supplementation. Some studies divided the intervention groups of children into more than one group to ascertain the effect of different combinations of probiotics or different concentrations on rotavirus diarrhoea. Five of these studies investigated the single-strain probiotic *Lactobacillus rhamnosus* GG [61,63,67,74,75]. *Saccharomyces boulardii* was investigated in six studies [58,60,64,65,66,72]. One aforementioned study [58] also investigated the effectiveness of *Lactiplantibacillus plantarum* LRCC5310 on rotavirus infection. Two other aforementioned studies [66,72] also investigated multi-strain probiotics. All the remaining studies investigated various multi-strain probiotics [55,59,62,68,69,70,71,73].

Two abstracts of additional studies in the English language were found [87,88] that noted a beneficial effect of the probiotics in the abstract, but a full text with all relevant data was not available despite contacting the authors; therefore, they were also not included in Table 1. Two studies [83,89] in the Chinese language also found a beneficial effect of probiotics for the prevention of diarrhoea in children, some of which tested positive for the rotavirus in stool samples; however, they were not included in Table 1 as only the abstract was in English. According to the abstracts, both studies found that probiotic supplementation with lactobacilli and/or bifidobacteria (species not specified in abstract) significantly decreased the incidence and duration of diarrhoea. Another study in the French language [90] also found an antiviral effect of *Saccharomyces cerevisiae* var. *boulardii* supplementation in children with acute diarrhoea (15 with rotavirus infection) and found a significant decrease in the duration of diarrhoea. The latter three mentioned studies were not included in Table 1 due to language barriers.

The effect of different multi-strain probiotics on rotavirus diarrhoea was significant after supplementation with *Bifidobacterium longum* BORI and *Lactobacillus acidophilus* AD031 [59], *Bifidobacterium longum* IBG, *Bifidobacterium lactis* BL, *Lactobacillus acidophilus* LA, *Lacticaseibacillus rhamnosus* LRH, *Lactiplantibacillus plantarum*, and *Pediococcus pentosaceus* [55], *Enterococcus faecalis* T-110, *Clostridium butyricum* TO-A and *Bacillus mesentericus* TO-A [62], unspecified strains of *Lactobacillus acidophilus*, *Lacticaseibacillus rhamnosus,* and *Saccharomyces boulardii* [66,68], VSL#3, containing four lactobacilli strains, three bifidobacteria strains, and one strain of *Streptococcus thermophilus* [69], BIFILAC, containing strains of *Enterococcus faecalis*, *Clostridium butyricum*, *Bacillus mesentericus* and *Bacillus coagulans* [70], Lakcid L, containing *Lacticaseibacillus rhamnosus* 573L/1, 573L/2, 573L/3 [71], *Lactobacillus casei and Lactobacillus acidophilus strains* CERELA [72] and *Lacticaseibacillus rhamnosus* 19070-2 and *L. reuteri* DSM 12,246 [73]. However, several studies did not report the strains used, which decreased the quality and reproducibility of the studies.

The probiotic strain *Lacticaseibacillus rhamnosus* GG, previously known as *Lactobacillus rhamnosus* GG (LGG), is a gram-positive lactobacillus, known to promote immunological responses and influence the intestinal microbiota by producing both a biofilm that can mechanically protect the mucosa, and different soluble factors beneficial to the gut by enhancing intestinal crypt survival, diminishing apoptosis of the intestinal epithelium, and preserving cytoskeletal integrity [91]. The ESPGHAN recommends LGG as an adjuvant therapy for gastrointestinal infections in children [77,80]. It was used in a large multi-centre European trial [74] with patients from Poland, Egypt, Croatia, Italy, Slovenia, the Netherlands, Greece, Israel, the United Kingdom, and Portugal. Administering the oral rehydration solution containing LGG to children with acute diarrhoea was found safe and resulted in a shorter duration of diarrhoea, less chance of a protracted course, and faster discharge from the hospital. There is also a large cohort of other studies using the same strain LGG that also confirms this effect [61,63,67,75]. A study by Szajewska et al. [92] that investigated the prevention of nosocomial diarrhoea found that supplementation with *Lacticaseibacillus rhamnosus* GG resulted in a reduced risk of nosocomial diarrhoea in children. A systematic review also confirms the reduction in the duration of rotavirus-induced diarrhoea, where a higher dose was efficient [93]. Another important factor to consider is the possible effect of rotavirus immunisation on the effectiveness of LGG, as noted in the meta-analysis [94], where the authors concluded that rotavirus immunisation affected the efficacy of LGG for the treatment of children with acute diarrhoea, which could be one of the underlying reasons for the mixed results. However, other reviews conclude that probiotics as adjuvants in vaccination should be considered in future studies, especially in the elderly and in children, where vaccine effectiveness and duration of immunisation really matter [38,95].

The probiotic yeast *Saccharomyces cerevisiae* var. *boulardii* is the only yeast used in clinical practice and is recommended for the prevention of antibiotic-associated diarrhoea and acute gastroenteritis in children as an adjunct [79,96] The mechanisms of action include inhibition of growth and invasion of pathogens by interfering with pathogen attachment, production of small peptides that inhibit endotoxins, as well as stimulation of short-chain fatty acids, especially butyrate, that restore intestinal functions and immunoregulation. However, the effect of *Saccharomyces cerevisiae* var. *boulardii* against common viruses responsible for diarrhoea, such as the rotavirus, adenovirus or norovirus, is still very limited, and further research is advocated [96]. *Saccharomyces cerevisiae* var. *boulardii* was efficient in the treatment of rotavirus gastroenteritis in children in six clinical studies noted in Table 1 [58,60,64,65,66,72].

In a small clinical study conducted in the Republic of Korea by Shin and co-authors [58], 50 hospitalized children with rotavirus enteritis were divided into three groups. The first group received a novel strain *Lactiplantibacillus plantarum* LRCC5310; however, neither the concentration of the probiotic nor the dosage was specified. Group II was the control group that did not receive any probiotics, and group III received a probiotic containing the *Saccharomyces cerevisiae* species according to the treatment policy of the hospital. Group III was retrospectively analysed through medical records. The novel strain LRCC5310 improved clinical symptoms and was comparable to, or more effective than the probiotic containing a *Saccharomyces cerevisiae* species. Several rotavirus genotypes were detected in stools, including: G9P8, G1P8, G1P18, G3P8, G2P4, G4P6, and G9P4. The rotavirus titre was significantly reduced in patients that received the novel strain LRCC5310 compared to those who did not take any probiotic formulations (Group II). Intake of LRCC5310 was found to be effective in the suppression of viral symptoms, as well as in prognosis and treatment, via virus titre reduction. The authors did not discuss the mechanisms involved, but the most likely mechanisms of the antiviral effect of the probiotic was due to modulation of the intestinal microbiota and the improvement of immune function, as several *Lactiplantibacillus plantarum* strains have exhibited enhancement of immune activity during infectious and inflammatory conditions, as well as improving lower gastrointestinal symptoms and modulation of intestinal microbiota after dysbiosis due to infections [97,98,99,100,101,102,103,104]. Although some probiotic traits are strain-specific, other core traits are in fact species-specific [31].

The study by Lee and co-authors [55] investigated the antiviral influence of a multi-strain probiotic against viral gastroenteritis in paediatric patients. Nine of the twenty-nine patients had a rotavirus infection. A six-species supplement containing *Bifidobacterium longum*, *Bifidobacterium lactis*, *Lactobacillus acidophilus*, *Lacticaseibacillus rhamnosus*, *Lactiplantibacillus plantarum*, and *Pediococcus pentosaceus* (strains not specified) proved effective in statistically significantly reducing the duration of diarrhoea in the probiotic group. Similarly, another multi-species probiotic, containing *Lactobacillus acidophilus*, *Lacticaseibacillus rhamnosus*, *Bifidobacterium longum,* and *Saccharomyces boulardii* (strains not specified) was also efficient [66,68]. Supplementation with bifidobacteria, including the probiotic *Bifidobacterium bifidum* Bb12, has been shown to protect against rotavirus infection, as children receiving this probiotic had a statistically significant lower concentration of the rotavirus-specific IgA antibody compared to the control group [105]. The well-known probiotic VSL#3 was also used in a study by Dubey et al. [69], conducted in India, and found a statistically significant lower duration and frequency of rotavirus diarrhoea in the probiotic group compared to the control group. Interestingly, the authors report that the statistically significant differences were still observed on day 4, but by day 8 the control group also spontaneously improved, and the results became comparable with the probiotic group. The antiviral effect of the multi-strain probiotic Bifilac was also found [70]. However, the author does not specify the composition of the supplement in the clinical trial.

Huang et al. [62] found that supplementation with a three-strain probiotic containing *Enterococcus faecalis* T-110, *Clostridium butyricum* TO-A, and *Bacillus mesentericus* TO-A resulted in a significant decrease in the duration of severe diarrhoea in the probiotic group compared to the placebo in children with infectious gastroenteritis. In the patients with rotavirus, a statistically significant decrease in gastroenteritis (Vesikari score) and diarrhoea frequency was also observed in the probiotic group. According to the authors of this study, the three strains acted symbiotically to facilitate the proliferation of the others. The dosage in this study was different compared to other clinical trials as the probiotic was given three times daily, whereas other clinical studies supplemented their patients once or twice a day.

Some of the clinical studies were not double-blind, but either single-blind [65,68] or open-labelled [61,62], which enhances the possibility of bias due to knowledge of the patient’s treatment group [106].

Besides probiotics, prebiotics [107], synbiotics [108], postbiotics [109], or even fermented foods [110] could have positive effects for rotavirus diarrhoea due to enhancement of the natural intestinal microbiota, as a combination of probiotics and prebiotics (synbiotics) could have a synergistic effect; in some cases, heat-killed probiotics or postbiotics could even be safer than viable microorganisms. Some human and animal studies have addressed these effects [84,111,112,113], opening the possibility for more well-designed clinical studies.

## 5. Studies with No Antiviral Effect of Probiotics against Rotavirus Infections

On the other hand, several other studies using the same strains or other strains did not find statistically significant differences after probiotic administration. *Lacticaseibacillus rhamnosus* GG did not appear to enhance short-term recovery following acute diarrhoeal illness in children in five clinical studies [114,115,116,117]. In a large study by Freedman et al. [118], no significant differences were found in paediatric patients aged between 3 and 48 months with diarrhoea after a five-day supplementation with 4.0 × 10^9^ cfu twice daily of either *Lacticaseibacillus rhamnosus* R0011 and *Lactobacillus helveticus* R0052 or *Lacticaseibacillus rhamnosus* GG, regardless of whether there were gastroenteritis-causing pathogens (e.g., adenovirus, norovirus, rotavirus, or bacteria). More results from the same clinical study (NCT01853124) were also published and showed no indication that probiotic administration lessened the burden of disease, regardless of the etiologic pathogen group (i.e., virus, bacteria, or parasite) or specific viral aetiologies (i.e., adenovirus, norovirus, or rotavirus) [119,120,121,122]. Perhaps the duration of supplementation with this probiotic was too short to exhibit a positive immunological effect as other clinical studies using the same strain achieved significant differences, such as Sindhu et al. [63] where the probiotic was consumed for 4 weeks, and an immunological effect was found in the probiotic group. Fewer children with rotavirus diarrhoea on LGG had repeated diarrhoeal episodes. Although no differences were found in the duration of diarrhoea, the immunological effect was evident. The dosage could also have been a factor, as Aggarwahl et al. and Basu et al. [61,67] both reported a statistically significant shorter duration of diarrhoea in children with watery diarrhoea after supplementation with LGG for five days at a dosage of 10^10^ CFU daily, whilst the dosage in the Freedman et al. study was 8.0 × 10^9^ CFU daily. No significant differences were also found in the immunogenicity of the rotavirus vaccine given to infants in a poor urban community in India after supplementation with the probiotic *Lacticaseibacillus rhamnosus* GG in a study by Lazarus et al. [123]; however, among probiotic recipients, the abundance of lactobacilli in stools showed a modest association with rotavirus shedding after the first dose of the vaccine, consistent with the concept that probiotic bacteria may promote vaccine virus replication and the immune response.

A study conducted in Vietnam [124] using *Lactobacillus acidophilus* (4.0 × 10^8^ CFU twice daily) also did not yield any significant differences in the duration of rotavirus diarrhoea compared to the placebo. The strain used was not specified and perhaps it was not a probiotic strain; or the dosage used was one log-step lower than other studies, which could have caused the lack of significant differences, as an appropriate strain and adequate administered amount of a probiotic are necessary to achieve a health benefit [31].

No significant differences were found in the studies conducted in Poland by Urbanska et al. [125] and Wanke et al. [126], using *Limosilactobacillus reuteri* DSM 17,938 for preventing nosocomial diarrhoea in children, including rotavirus infection. *Limosilactobacillus reuteri* DSM 17,938 has otherwise shown to be effective in the prevention and treatment of infantile colic and regurgitation and gastrointestinal disorders [127,128,129].

Several other clinical studies also did not show a statistically significant anti-rotavirus effect after intervention with *Bacillus coagulans* [130], *Lacticaseibacillus casei* ST11 [131]. A study using *Bifidobacterium animalis* subsp. *lactis* Bb-12 and *Streptococcus thermophilus* TH4 also did not find any differences in the treatment of gastroenteritis compared to the placebo; however, a decrease in rotavirus shedding was observed [86].

One of the possible reasons for the lack of effect of probiotics in these clinical trials, even though the same strains were used that previously exhibited a health benefit, could have been the quality of the production procedures of the probiotic strains. Lyophilisation, and the form of probiotics including lyophilized or heat-dried powders in capsule or powder form, can also influence the shelf-life and general quality of the probiotic [132]. Depending on production, some probiotics need to be stored in the refrigerator and others do not. Finally, the stability of the product must remain during storage, as an adequate concentration of viable probiotics must be persevered for the whole shelf-life. The probiotics used in clinical studies come from various commercial markets, and since many are foodstuffs or dietary supplements—not medicinal products—the quality may not always be assured or controlled [132,133]. All these factors can indirectly influence the reality of the results of clinical studies.

## 6. Conclusions, Limitations, and Future Directions

The effect of probiotics on enteric virus infections has been studied for years, and there is still much research to be done in the line of the microbiota–host–pathogen interactions. Although probiotics have shown promising results in the prevention of viral AGE, we still need an effective weapon to prevent the high mortality rate in early childhood in low-income countries. At least for rotavirus infection, effective vaccines are available to make progress in lowering the disease burden.

In probiotics studies, we need to be cautious as results are inconsistent—sometimes the same probiotic strain was not effective, whilst in other clinical studies it was, showing the importance of correct dosages, the duration of treatment, and quality of the probiotic.

With careful consideration of strains, dosages, and durations of supplementation, probiotics appear to be a safe and effective adjuvant in the treatment of rotavirus diarrhoea via modulation of the immune system and the intestinal microbiota. However, more clinical studies with different probiotics, perhaps combined with prebiotics to achieve a synergistic effect to optimally influence the restoration of the intestinal microbiota after dysbiosis due to diarrhoea, are warranted.

## Figures and Tables

**Table 1:** Characteristics of 19 clinical studies with statistically significant antiviral effects of probiotics against rotaviruses.

Reference (First Author, Year) ^1^	Study Design	Population	Intervention			Main Findings
			Active	Control	Duration	
Shin et al. (2020), Rep. Korea [58]	RCT	50 hospitalized children with rotavirus gastroenteritis, aged up to 6 years. 15 in novel probiotic group. 8 in control group (group II). 27 in group III (retrospectively analysed through medical records).	Group I: *Lactiplantibacillus*^3^*plantarum* LRCC5310 Group III: Probiotic *Saccharomyces cerevisiae* species ^2^ according to hospital treatment policy Dosage: not specified.	Group II: standard treatment	Up to 8 days	Group I (*Lactiplantibacillus plantarum* LRCC5310) showed a statistically significant improvement in the number of patients with persistent diarrhoea, number of defecation events per day, and total diarrhoea period compared to group II (control). Group I showed slight improvement in the number of patients with loose stools, number of defecation events, and diarrhoea duration compared to group III (*Saccharomyces cerevisiae*-containing probiotic formulation).
Park, Kwon, Ku, and Ji (2017), Korea [59]	Double-blind RCT	57 hospitalized infants with rotavirus disease, aged between 9 and 16 months. 28 in probiotic group. 29 in control group.	*Bifidobacterium longum* BORI *Lactobacillus acidophilus* AD031 Dosage: 2.2 × 10^9^ cfu/g twice daily	Placebo	3 days	A significantly shorter duration of patients’ diarrhoea was observed in the probiotic group compared to the placebo group. Symptoms such as duration of fever, frequency of diarrhoea, and frequency of vomiting tended to be ameliorated by the probiotic treatment; however, differences were not statistically significant between the two groups.
Das, Gupta, and Das (2016), India [60]	Double-blind RCT	60 children, aged between 3 months to 5 years, with watery diarrhoea and stool rotavirus positive. 30 in probiotic group. 30 in control group.	*Saccharomyces cerevisiae* var. *boulardii* Dosage: 250 g sachets twice daily	Placebo	5 days	A significantly shorter duration of diarrhoea and hospitalization was observed in the intervention group. No significant difference was seen for fever and vomiting. There was also no difference between the two groups in the proportion of children requiring parenteral rehydration and persistence of diarrhoea lasting beyond day 7.
Lee et al. (2015), Rep. Korea [55]	Double-blind RCT	29 children between 3 months and 7 years with viral gastroenteritis (9 rotavirus infection). 13 in probiotic group. 16 in control group.	*Bifidobacterium longum* IBG, *Bifidobacterium lactis* BL, *Lactobacillus acidophilus* LA, *Lacticaseibacillus*^3^*rhamnosus* LRH, *Lactiplantibacillus*^3^*plantarum*^2^, *Pediococcus pentosaceus*^2^ Dosage: 10^9^ cfu/g twice daily.	Placebo	1 week	The multi-strain probiotic significantly shortened the duration of diarrhoea and fever compared to the placebo. The mean duration of vomiting was shorter in the probiotic group, but the difference in the study groups was not statistically significant.
Aggarwal et al. (2014), India [61]	Open Label RCT	200 children with watery diarrhoea (41 positive for rotavirus in stool), aged between 6 months and 5 years. 100 in probiotic group. 100 control group.	Culturelle probiotic GG contains: *Lacticaseibacillus*^3^*rhamnosus* GG (LGG) Dosage: 10^10^ cfu/g once daily	Standard treatment	5 days	A statistically significant decrease in the duration of diarrhoea, faster improvement in stool consistency, and reduction in average number of stools per day was observed in the probiotic group compared to standard treatment.
Huang et al. (2014), Taiwan [62]	Open Label RCT	159 hospitalized children with infectious gastroenteritis (42 rotavirus), aged between 3 months to 14 years. 82 in probiotic group. 77 in control group.	Bio-three contains ^4^: *Enterococcus faecalis* T-110, *Clostridium butyricum* TO-A, *Bacillus mesentericus* TO-A Dosage: 3.48 × 10^8^ cfu/g 3 times daily	standard treatment	7 days	A statistically significant decrease in the duration of severe diarrhoea was observed in the probiotic group compared to standard treatment. In the patients with rotavirus, a statistically significant decrease in gastroenteritis (Vesikari score) and diarrhoea frequency was also observed in the probiotic group.
Sindhu et al. (2014), India [63]	Double-blind RCT	124 children with gastroenteritis, aged between 6 months to 5 years, infected either with rotavirus (82) or *Cryptosporidium* species (42). 65 in probiotic group. 59 in control group.	*Lacticaseibacillus*^3^*rhamnosus* GG Dosage: 10^10^ cfu/g once per daily	Placebo	4 weeks	A statistically significant increase in the IgG levels post-intervention was observed in children with rotavirus diarrhoea receiving LGG after 4 weeks. Fewer children with rotavirus diarrhoea on LGG had repeated diarrhoeal episodes. No differences were found in duration of diarrhoea.
Corrêa, Penna, Lima, Nicoli, and Filho (2011), Brazil [64]	Double-blind RCT	186 hospitalized children (57.4% with rotavirus), aged between 6 to 48 months, with acute diarrhoea. 90 in probiotic group. 86 in control group.	*Saccharomyces cerevisiae* var. *boulardii* Dosage: 4 × 10^9^ cfu/g twice daily	Placebo	5 days	A statistically significant reduction in the duration of diarrhoea was observed in probiotic group compared to placebo.
Dalgic, Sancar, Bayraktar, Pullu, and Hasim (2011), Turkey [65]	Single Blind RCT	240 children with rotavirus diarrhoea, divided into eight groups. 60 in each group.	Group 1: *Saccharomyces cerevisiae* var. *boulardii*, Group 2: zinc (Zn), Group 3: lactose-free formula (LF), Group 4: Zn and *Saccharomyces boulardii* (SB), Group 5: LF and *Saccharomyces boulardii* (SB), Group 6: Zn and LF, Group 7: Zn and LF and *Saccharomyces boulardii* (SB), Group 8: control Dosage: 250 mg once daily	Standard treatment	5 days	A statistically significant reduction in diarrhoea duration and hospital stay was observed in groups 2 and 4 compared to standard treatment. A significant difference in the duration of hospitalization between groups 1 and 4; groups 2 and 7; groups 3 and 4; groups 4 and 5, and groups 4 and 7 was also found.
Grandy, Medina, Soria, Terán, and Araya (2010), Bolivia [66]	Double-blind RCT	64 hospitalized children with rotavirus infection, aged 1 to 23 months, divided in 3 groups. Group 1: single strain probiotic (20). Group 2: multi-strain probiotic (23). Group 3: control (21)	Group 1: *Saccharomyces cerevisiae* var. *boulardii* Dosage: 4 × 10^10^ cfu/g twice daily Group 2: *Lactobacillus acidophilus*^2^, *Lacticaseibacillus*^3^*rhamnosus*^2^, *Bifidobacterium longum*^2^ *Saccharomyces cerevisiae* var. *boulardii* Dosage: 1.25 × 10^8^ cfu/g twice daily	Placebo	5 days	Statistically significant decrease in duration of diarrhoea shorter duration of fever was observed in children who received the single-species probiotic compared to the placebo. Statistically significant fewer episodes of vomiting were observed with the multi-species probiotic compared to the placebo. When probiotic groups were merged, the statistical significance of changes increased (total duration of diarrhoea, fever, and vomiting).
Basu, Paul, Ganguly, Chatterjee, and Chandra (2009), India [67]	Double-blind RCT	559 hospitalized children (319 with rotavirus), aged up to 2 years, divided into 3 groups. 185 in group A. 188 in group B. 186 in group C.	Group A: control Group B: LGG, Dosage: 10^10^ cfu/g twice daily. Group C: LGG, Dosage: 10^12^ cfu/g twice daily	Standard treatment	7 days	A statistically significant lower frequency and the duration of diarrhoea, requirement for intravenous therapy, and hospital was observed in both the intervention groups compared with the control. There was no significant difference between the 2 intervention groups.
(Teran, Teran-Escalera, and Villarroel (2009), Bolivia [68]	Single Blind RCT	75 hospitalized children, aged from 28 days to 24 months with rotavirus diarrhoea, divided into three groups. Group 1: nitazoxanide (25). Group 2: probiotic group (25). Group 3: control (25).	Group 1: nitazoxanide Group 2: *Lactobacillus acidophilus*^2^, *Lacticaseibacillus*^3^*rhamnosus*^2^, *Bifidobacterium longum*^2^ and *Saccharomyces cerevisiae* var. *boulardii* Dosage: 1.25 × 10^8^ cfu/g twice daily	Standard treatment	5 days	A statistically significant reduction in the duration of diarrhoea and hospital stay was observed in the probiotic group compared to standard treatment.
Dubey, Rajeshwari, Chakravarty, and Famularo (2008), 2008, India [69]	Double-blind RCT	230 hospitalized children with rotavirus diarrhoea, aged between 6 months and 2 years. 113 in probiotic group. 111 in control group.	VSL#3 contains ^4^: 4 strains of lactobacilli species: *Lactobacillus acidophilus*, *Lacticaseibacillus*^3^*paracasei*, *Lactobacillus bulgaricus*, *Lactiplantibacillus*^2^*plantarum*, 3 strains of Bifidobacteria species: *Bifidobacterium breve*, *Bifidobacterium infantis*, *Bifidobacterium longum*, 1 strain of Streptococcus thermophilus Dosage: 9 × 10^10^ cfu/g twice daily	Placebo	4 days	A statistically significant lower mean stool frequency and improved stool consistency was observed after day 2 up to day 4. After day 4, the control group also showed spontaneous improvement. The overall recovery rates were significantly better in the probiotic group compared with the placebo. A statistically significant lower overall requirement for oral rehydration salts was found.
Narayanappa (2008), India [70]	Double-blind RCT	80 hospitalized children with rotavirus diarrhoea, aged between 3 months and 3 years. 40 in probiotic group. 40 in control group.	BIFILAC contains ^4^: *Enterococcus faecalis*^2^, *Clostridium butyricum*^2^, *Bacillus mesentericus*^2^, *Bacillus coagulans*^2^ Dosage: 1 sachet three times daily	Placebo	Up to 14 days	A statistically significant reduction in the number of episodes (frequency) of diarrhoea, mean duration of diarrhoea, degree of dehydration, duration and volume of oral rehydration salt therapy, duration and volume of intravenous fluid therapy and duration of rotavirus shedding was observed in the probiotic group compared to the control group.
Szymański, Pejcz, et al. (2006), Poland [71]	Double-blind RCT	87 children with infectious diarrhoea (39 with rotavirus), aged between 2 months and 6 years. 49 in probiotic group. 44 in control group.	Lakcid L contains: *Lacticaseibacillus*^3^*rhamnosus* 573L/1, *Lacticaseibacillus*^3^*rhamnosus* 573L/2 *Lacticaseibacillus*^3^*rhamnosus* 573L/3 Dosage: 1.2 × 10^9^ cfu/g twice daily	Placebo	5 days	A statistically significant reduction in the duration of rotavirus diarrhoea, but not of diarrhoea of any aetiology, in children was observed in the probiotic group compared to the control group. Intervention shortened the time of intravenous rehydration.
Gaón et al. (2003), Argentina [72]	Double-blind RCT	89 hospitalized children with infectious diarrhoea (27% with rotavirus), aged between 6 and 24 months. 29 in control group (group 1). 30 in group 2. 30 in lactobacilli group 3.	Group 1: placebo Group 2: *Saccharomyces cerevisiae* var. *boulardii*, Group 3: *Lacticaseibacillus*^3^*casei* and *Lactobacillus acidophilus* CERELA Dosage: 10^10^–10^12^ cfu/g twice daily	Placebo	5 days	A statistically significant reduction in the duration of diarrhoea and number of stools in children was observed in all probiotic groups compared to the control group.
Rosenfeldt et al. (2002), Denmark [73]	Double-blind RCT	69 hospitalized children with infectious diarrhoea (66% with rotavirus), aged between 6 and 36 months. 30 in probiotic group. 39 in control group.	*Lacticaseibacillus*^3^*rhamnosus* 19070-2, *Limosilactobacillusreuteri* DSM 12246, Dosage: 2.2 × 10^10^ cfu twice daily.	Placebo	5 days	A statistically significant reduction in the duration of hospital stay was observed in the probiotic group compared to the placebo. The beneficial effects (duration of diarrhoea, loose stool, length of hospital stay) were most prominent in children treated early in the diarrhoeal phase.
Guandalini et al., (2000), European study [74]	Double-blind RCT	287 children with liquid or semiliquid stools (101 with rotavirus), aged between 1 month and 3 years. 147 in probiotic group. 140 in placebo group.	*Lacticaseibacillus*^3^*rhamnosus* GG, Dosage: 10^10^ cfu in 250 mL of standard treatment solution. Solution added to patient according to need.	Standard treatment	Up to 7 days	A statistically significant reduction in duration of diarrhoea and duration of hospital stay in rotavirus-positive and rotavirus-negative children was observed in the probiotic group compared to the control group. In rotavirus-positive children, a significant reduction in number of average stools was also found in the probiotic group compared to the control group.
Guarino, Canani, Spagnuolo, Albano, and Di Benedetto (1997), Italy [75]	Double-blind RCT	100 children with diarrhoea (61 positive for rotavirus), aged between 3 and 36 months. 52 in probiotic group. 48 in control group.	*Lacticaseibacillus*^3^*rhamnosus* GG ^5^, Dosage: 10^10^ cfu in 200 m twice daily	Standard treatment	Up to 5 days	A statistically significant reduction in the duration of diarrhoea rotavirus-positive and rotavirus-negative ambulatory children with diarrhoea was observed in the probiotic group compared to the control group. Furthermore, the duration of rotavirus excretion was reduced.

^1^ Clinical studies in descending chronological order, arranged alphabetically. RCT: Randomised, controlled trial. ^2^ Strain not specified. ^3^ Nomenclature of species has been updated according to Zheng et al., 2020 [76], ^4^ information of strains not reported in published clinical trial but retrieved from public website. ^5^ The clinical trial incorrectly notes the probiotic as *Lactobacillus casei* GG.

## Data Availability

Not applicable.
